# Retraction: Ball flower like manganese, strontium substituted hydroxyapatite/cerium oxide dual coatings on the AZ91 Mg alloy with improved bioactive and corrosion resistance properties for implant applications

**DOI:** 10.1039/d3ra90029k

**Published:** 2023-03-29

**Authors:** D. Gopi, N. Murugan, S. Ramya, E. Shinyjoy, L. Kavitha

**Affiliations:** a Department of Chemistry, Periyar University Salem 636 011 Tamilnadu India dhanaraj_gopi@yahoo.com +91 427 2345124 +91 427 2345766; b Centre for Nanoscience and Nanotechnology, Periyar University Salem 636 011 Taminadu India; c Department of Physics, School of Basic and Applied Sciences, Central University of Tamilnadu Thiruvarur 610 101 Tamilnadu India

## Abstract

Retraction of ‘Ball flower like manganese, strontium substituted hydroxyapatite/cerium oxide dual coatings on the AZ91 Mg alloy with improved bioactive and corrosion resistance properties for implant applications' by D. Gopi *et al.*, *RSC Adv.*, 2015, **5**, 27402–27411, DOI: https://doi.org/10.1039/C5RA03432A.

The Royal Society of Chemistry, with the agreement of the named authors, hereby wholly retracts this *RSC Advances* article due to concerns with the reliability of the data in the published article.

The optical images presented in Fig. 6a and c are identical. Furthermore, the images in Fig. 6a, c and e have been duplicated in other publications. The panels in Fig. 6a and c have been duplicated as Fig. 9a in ref. 1, and as Fig. 8a in ref. 2. The panel in Fig. 6e has been duplicated as Fig. 9b in ref. 1, and as Fig. 8b in ref. 2.

Part of the image in Fig. 10c has been duplicated in Fig. 10e.

The authors informed the Editor that the characterization of the original samples was outsourced, and they do not have the original raw data for the published results.

Given the significance of the concerns about the validity of the data, and the lack of raw data, the findings presented in this paper are not reliable.

N. Murugan was contacted but did not respond.

Signed: D. Gopi, S. Ramya, E. Shinyjoy and L. Kavitha

Date: 16th March 2023

Retraction endorsed by Laura Fisher, Executive Editor, *RSC Advances*

Retractions

## Supplementary Material
